# Bioresorbable scaffolds: a new paradigm in percutaneous coronary intervention

**DOI:** 10.1186/s12872-016-0207-5

**Published:** 2016-02-12

**Authors:** Erhan Tenekecioglu, Vasim Farooq, Christos V. Bourantas, Rafael Cavalcante Silva, Yoshinobu Onuma, Mustafa Yılmaz, Patrick W. Serruys

**Affiliations:** ThoraxCentre, Erasmus Medical Centre, Rotterdam, The Netherlands; Manchester Heart Centre, Manchester Royal Infirmary, Central Manchester University, Hospitals NHS Trust, Manchester, UK; Institute of Cardiovascular Sciences, University College of London, London, UK; Department of Cardiology, Barts Health NHS Trust, London, UK; Department of Cardiology, Bursa Postgraduate Education and Research Hospital, Bursa, Turkey; International Centre for Circulatory Health, Imperial College, London, UK; Interventional Cardiology Department, Erasmus MC, ‘s-Gravendijkwal 230, Rotterdam, 3015 CE The Netherlands; Institute of Cardiovascular Sciences, Manchester Academic Health Sciences Centre, University of Manchester, Manchester, UK

**Keywords:** Bioresorbable scaffolds, Cronary artery disease, Coronary stents

## Abstract

Numerous advances and innovative therapies have been introduced in interventional cardiology over the recent years, since the first introduction of balloon angioplasty, but bioresorbable scaffold is certainly one of the most exciting and attracting one. Despite the fact that the metallic drug-eluting stents have significantly diminished the re-stenosis ratio, they have considerable limitations including the hypersensitivity reaction to the polymer that can cause local inflammation, the risk of neo-atherosclerotic lesion formation which can lead to late stent failure as well as the fact that they may preclude surgical revascularization and distort vessel physiology. Bioresorbable scaffolds overcome these limitations as they have the ability to dissolve after providing temporary scaffolding which safeguards vessel patency. In this article we review the recent developments in the field and provide an overview of the devices and the evidence that support their efficacy in the treatment of CAD. Currently 3 devices are CE marked and in clinical use. Additional 24 companies are developing these kind of coronary devices. Most frequently used material is PLLA followed by magnesium.

## Background

### The need for Bioresorbable scaffolds

Plain ‘old’ balloon angioplasty (POBA) was first performed by Andreas Roland Grüntzig in 1977 and heralded the first revolution in the percutaneous treatment of coronary artery disease (CAD) [1]. Despite the success in dilating and restoring coronary flow to diseased coronary vessels, enthusiasm to this ground-breaking technology was hampered by issues related to acute vessel closure secondary to iatrogenic coronary dissection (occurring in approximately 30–40 % of cases) and restenosis secondary to elastic re-coil, constrictive remodelling, and neointimal hyperplasia [2–5]. Bare metal stents (BMS) heralded the second revolution in the treatment of CAD as means to overcome the limitations of POBA. BMS resolved the issue of acute vessel occlusion by sealing the dissection flaps and prevented elastic recoil and constrictive remodelling. Two landmark studies - BENESTENT and STRESS trials - demonstrated the superiority of bare metal stents (BMS) over POBA [6–8]. Nevertheless, indigenous limitations of BMS such as the neointimal hyperplasia and consequent the increased risk of in-stent restenosis (ISR) precluded the widespread adoption of this technology, particularly in more complex CAD and diabetics [9–12].

Drug eluting stents (DES) – the third revolution in interventional cardiology – were conceived as means to tackle the iatrogenic issue of excessive neointimal hyperplasia and reduce the risk of restenosis. Land-mark studies of the first generation sirolimus-eluting Bx velocity stents demonstrated the dramatic reduction in the excessive hyperplastic healing response and risk of restenosis compared to BMS [13, 14]. Subsequently the indications for DES rapidly expanded, with the use of DES in more complex CAD and higher risk patient groups. Despite the promising results associated with the first generation DES, safety issues arose, in particular the risk of late stent thrombosis, quoted as 0.53 % per year, with a cumulative incidence of 3.3 % at 4 years [15, 16]. The primary concerns with the first generation DES were related 1) to the lack of biocompatibility of the drug eluting polymer leading to a persistent inflammatory response beyond the drug eluting period of the device, 2) to a risk of a continued neointimal response and risk of a ‘late-catch up’ phenomenon and late ISR, and 3) to a delayed/incomplete healing, and risk of late/very late stent thrombosis. In addition, other issues were identified including, stent malapposition (early or late acquired), the risk of early or late stent fracture, neoatherosclerotic lesion formation and late DES failure, and the permanent metallic caging causing abnormal vasomotion [17]. With the latter, abnormal vasoconstriction responses to acetylcholine at the sites distal to the DES were identified, implying the abnormal function of the endothelial layer. Although newer generation DES, with more biocompatible polymers, overcame many of the safety issues related to first generation DES, these concerns were not completely resolved especially the longer term risk of DES failure secondary to neoatherosclerosis [18–20]. Bioresorbable scaffolds (BRS) – heralded as the fourth revolution in interventional cardiology – were thus designed to overcome the perceived limitations of DES by providing a temporary support to the vessel wall, whilst simultaneously allowing for the release of an anti-proliferative drug to limit the excessive response, in order to potentially allow the vessel to heal and restore its physiological function.

### Development of bioresorbable scaffolds

Historically biodegradable materials for implants which serve as a temporary function have been used in therapeutic medicine in areas that include wound closure – such as absorbable surgical sutures made from glycolic and lactic acid orthopaedic devices, dental procedures, cardiovascular surgery, intestinal surgery, urology, nerve repair, drug delivery and oncology, and were designed to overcome the disadvantages of permanent metallic-based devices [21].

In so far as application with BRS, this concept is still in its infancy. Identifying the appropriate bioresorbable materials to allow for temporary scaffolding of the vessel wall to seal dissections and prevent recoil, and allowing for drug elution to limit the healing response has proven to be a major challenge. In addition, the ideal BRS should have as thin struts as possible to limit the healing response whilst providing adequate radial support for a 3–6 month period to limit recoil and constrictive remodelling, and have as low crossing profile as possible and be flexible enough to allow delivery in more challenging anatomical disease.

Various types of materials have been used in BRS development (Table 1). Amongst them poly-L-lactic acid (PLLA) and magnesium appear to be the most promising and reached clinical use. PLLA is the most commonly used material for manufacturing BRS. The degradation of PLLA is by hydrolysis of the ester bonds into small particles that are phagocytosed by macrophages into lactic acid and metabolized through the Krebs cycle into carbon dioxide and water [22]. Magnesium is mixed with rare earth metals to allow it to have thinner struts and control the degradation process. In addition magnesium has been reported to have potential antithrombotic properties emanating from its electronegative charge during degradation [23, 24]. One of the reported challenges associated with magnesium alloys has been the too rapid degradation of the material before the end of the healing process with the consequent risk of early vessel recoil and restenosis [25].

**Table 1:** Summary of the design and structure of clinically tested bioresorbable scaffolds

Scaffold	Strut material	Coating material	Eluted drug	Strut thickness (μm)	Resorption (month)	Current status
Igaki-Tamai	PLLA	None	None	170	24–36	CE mark for peripheral use
AMS-1	Mg	None	None	165	<4	Discontinued
DREAMS-1	Mg	PLGA	Paclitaxel	125	9	Clinical trials
DREAMS-2	Mg	PLLA	Sirolimus	150	9	Clinical trials
Absorb BVS 1.0	PLLA	PDLLA	Everolimus	156	18–24	Discontinued
Absorb BVS 1.1	PLLA	PDLLA	Everolimus	156	24–48	CE mark
Absorb BVS-New generation	PLLA	PDLLA	Everolimus	<100	NA	NA
DeSolve	PLLA	None	Myolimus	150	12–24	CE mark
DeSolve 100	PLLA	PLLA	Novolimus	100	24	CE mark
IDEAL biostent	Polymer salicylate	Salicylate	Sirolimus	175	>12	Clinical trials
REVA	PTD-PC	None	None	200	24	Discontinued
ReZolve	PTD-PC	None	Sirolimus	115–230	4–6	Clinical trials
ReZolve2	PTD-PC	None	Sirolimus	100	48	Clinical trials
Fantom	PTD-PC	-	Sirolimus	125	36	Clinical trials
Fortitude	semicrystalline polylactide	-	None	150–200	3–6	Clinical trials
Mirage BRMS	PLLA	-	Sirolimus	125–150	14	Clinical trials
MeRes	PLLA	PDLLA	Sirolimus	100	24	Clinical trials
Xinsorb	PLLA	PDLLA	Sirolimus	160	24–36	Clinical trial
ART 18AZ	PDLLA	None	None	170	3–6	Clinical trials

*Mg* magnesium, *PLLA* poly-L-lactic acid, *PDLLA* poly-DL-lactic acid, *BVS* bioresorbable vascular scaffold

*SA*/*AA* salicylic acid/adipic acid, *PTD*-*PC*, poly-tyrosine-derived polycarbonate, *CE* Conformité

Européenne. *NA* not available

### The potential benefits of bioresorbable scaffolds

BRS allow for successful acute revascularization of coronary artery stenosis and in preliminary studies, they have been shown to be associated with low rates of repeat revascularisation and major adverse cardiac events (MACE) during the early follow-up period [26]. The main advantage of the BRS is that following complete bioresorption, no foreign body remains in the vessel wall at long term follow-up, which may mitigate the increased long term risk of stent thrombosis seen with the first generation DES [22, 27, 28]. In addition, a potential issue of late catch-up in restenosis secondary to a persistent low grade inflammatory response to the polymer/device, even evident with newer generation DES [29], may be mitigated with BRS since no material remains following bioresorption.

The enhanced mechanical flexibility of the Absorb BRS (compared to metallic DES) allows for increased conformability to the original vessel wall geometry, which may have an advantageous influence on coronary blood flow and its biomechanical properties [30]. Additionally the bioresorption process allows for malapposed struts or jailed struts over the side-branch to resolve at follow-up [30]. Furthermore, the treated vessel has been shown to potentially restore its vasomotor function a year following Absorb BRS implantation, when the structural integrity of the Absorb device has been appropriately lost [31]. Conversely, endothelial dysfunction has been shown to persist with DES [31]. Another useful property of BRS is that it allows for a non-invasive imaging (e.g. multi-slice coronary computed tomography [MSCT]) without any significant imaging artefacts. Additionally, BRS potentially allows the surgeons to attach anastomoses to the scaffolded segments once the bioresorption process has been completed.

### Bioresorbable scaffolds currently in use

#### Absorbable magnesium stent (AMS)

**Fig. 1:**
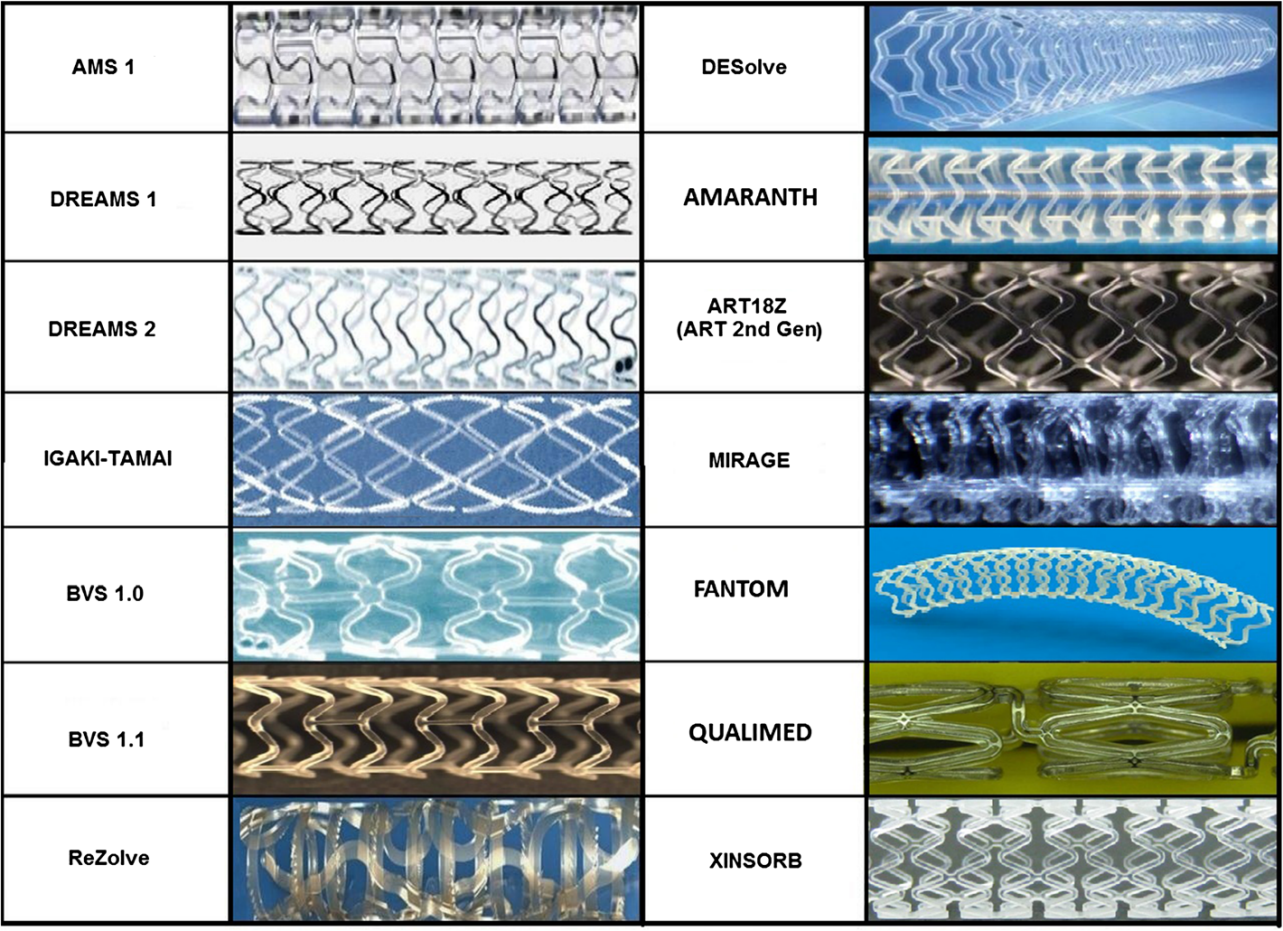
Design of bioresorbable scaffolds in clinical or preclinical use

**Fig. 2:**
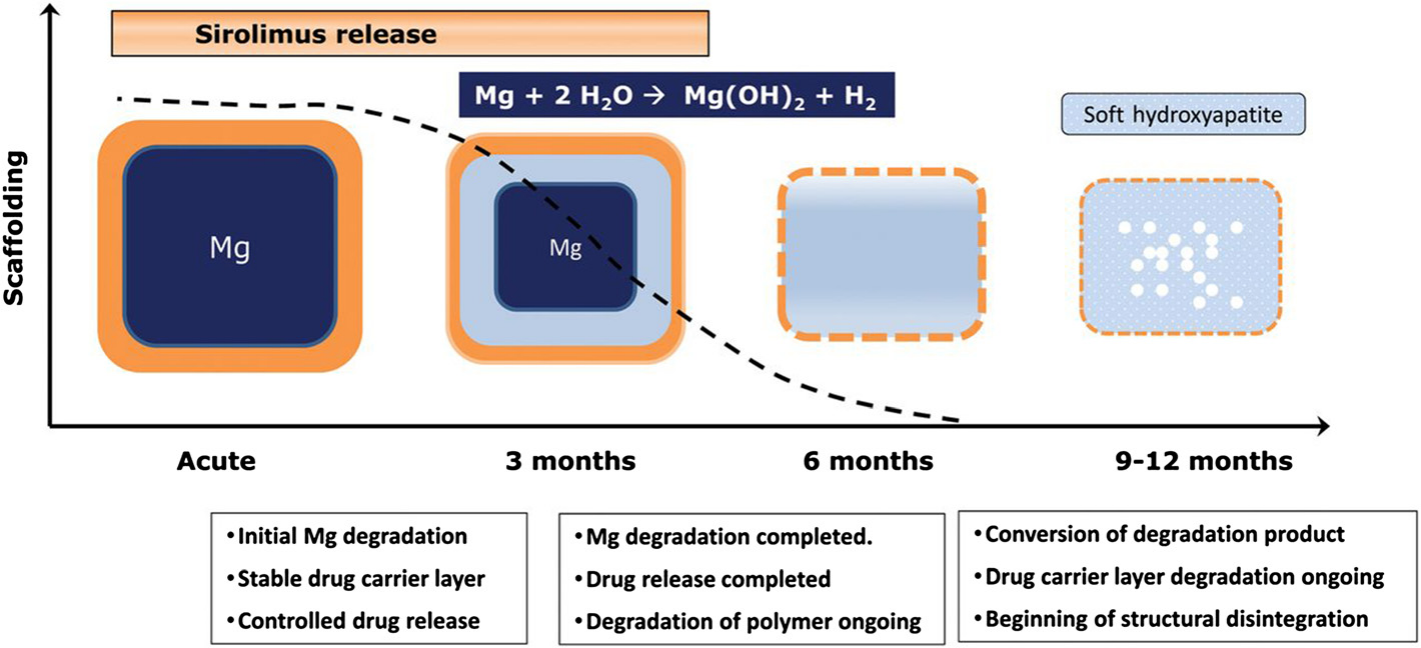
Device functionality of drug-eluting absorbable metal scaffold over time. (Reprinted from European Heart Journal with permission from Oxford University Press)

Magnesium (Mg) is an essential element for several enzymes in human body and a co-factor for ATPase. The balloon-expandable Absorbable Metal Stent (AMS-1) (Biotronik, Berlin, Germany) (Fig. 1) was the first metallic bioresorbable scaffold. The radial strength of the device allowed for low elastic recoil (<8 %), a high collapse pressure (0.8 bar), and minimal shortening after inflation (<5 %) [32]. In preclinical studies, rapid endothelization of the device and degradation into inorganic salts was reported within 60 days [33, 34] (Fig. 2). In the prospective, multicentre, non-randomized, Clinical Performance and Angiographic Results of Coronary Stenting with Absorbable Metal Stents (PROGRESS AMS) pilot study (63 patients with single de novo lesions, 71 AMS), no death, MI or stent thrombosis was reported at 12 months follow-up, with the treated vessel attaining its vasoreactivity within 4-months. The device was however associated with an unacceptable incidence of repeat revascularisation (target lesion revascularization [TLR] rates 23.8 % and 45 % at 4 months and 12 months respectively), which was similar to POBA [32]. Intravascular ultrasound (IVUS) imaging revealed that the late lumen loss (LLL) (1.08 mm at 4-months) was due to recoil secondary to inadequate radial force that was ascribed to be secondary to the too rapid degradation of the device. Afterwards the device was redesigned predominantly to slow down the bioresorption process, so as to retain its mechanical strength for longer in order to prevent early vessel recoil. Several design iterations have emerged: AMS-2 and AMS-3. The AMS-2 scaffold had a more refined Mg alloy which gave it a higher collapse pressure (1.5 bar compared with 0.8 bar for AMS-1), approximately 30 % thinner struts (from 165 μm [AMS 1] to 125 μm), and importantly, a longer bioresorption process – with a 2–3 times slower degradation process. The AMS-3 ‘DREAMS’ (Drug Eluting Absorbable Metal Scaffold) device uses a similar platform as the AMS-2, and includes a biodegradable polymer that allows for drug elution. The DREAMS device provides vessel scaffolding and paclitaxel drug elution for a period of 3 months (Fig. 1). DREAMS was tested in clinical setting in the BIOSOLVE-I study [35]. In this prospective, multi-center, non-randomised trial, 46 patients with a single de novo lesion with a reference diameter 3.0–3.5 mm were recruited. In total, 47 DREAMS devices were successfully implanted. At 6-months the TLR rate was 4.3 % and the LLL was 0.64 ± 0.50 mm. At this same time point, improvements in the scaffolded segment angulation were evident, from 14.9 ± 12.0° post-procedurally, to 26.1 ± 15.9° at follow-up [35]. Further modification of the DREAMS device alloy - made from a WE43 alloy with 6-crown 2-link design - have allowed for a slower bioresorption and dismantling process. The DREAMS-2 device has a strut thickness of 150 μm and incorporates tantalum based radiopaque markers at both ends to allow for more precise post-dilatation. In addition, the DREAMS-2 device was coated with a bioresorbable polylactic acid polymer (7 μm) and a limus based anti-proliferative drug (sirolimus at a dose of 1.4 μg/mm^2^) – which was shown to have a more potent anti-proliferative effect compared to paclitaxel. DREAMS-2 has completed preclinical evaluation and is currently being investigated in the BIOSOLVE-II study (*n* = 120).

### Polymeric scaffolds

#### The igaki-tamai scaffold

The Igaki-Tamai scaffold (Kyoto Medical Planning Co., Ltd., Kyoto, Japan) was the first BRS used in humans, and is a PLLA-based, non-drug eluting and a heat treated self-expandable device [36]. For the initial expansion of the device, the contrast was heated up to 80 °C and applied through the delivery balloon. Final expansion of the device was achieved at body temperature after 20–30 min following device implantation. In vivo the device took 18–24 months to fully disappear. To allow for visualization during the follow-up, two radiopaque cylindrical gold markers were placed at both ends of the device. A pilot study examining the efficacy of this device (15 patients, 19 lesions, 25 stents), demonstrated no MACE or ST within 30 days and only 1 repeat PCI at the 6-month follow-up. The mean stent cross-sectional area increased from 7.42 ± 1.51 mm^2^ at baseline to 8.18 ± 2.42 mm^2^ (*P* = 0.086) at 3 months, and 8.13 ± 2.52 mm^2^ at 6 months follow-ups (*p* =0.30) [36]. Notably, there was no significant neo-intimal hyperplasia on IVUS. IVUS also demonstrated no significant stent recoil at day-1 but evidence of stent expansion at 3-months following implantation.

In a second study of 50 elective patients (63 lesions, 84 stents), IVUS follow up at 3-year demonstrated complete absence of the struts. In addition, angiographic mean diameter stenosis was 25 % compared to 38 %, 29 %, and 26 % at 6, 12, and 24 months, respectively. At 4-year follow-up, the overall and MACE-free and survival free rates were 97.7 % and 82.0 % respectively [37]. Ten-year clinical follow-up demonstrated freedom from cardiac death, non-cardiac death, and MACE at 98 %, 87 %, and 48 %, respectively [38]. Angiographic long term follow-up demonstrated no changes in the minimal lumen diameter (MLD): 1-year mean MLD 2.01 mm; 10-year mean MLD 2.06 mm. Only 2 ST events were reported at 10-year follow-up. Concerns with regards to this device arose from the use of heat to induce self-expansion, which may cause arterial wall necrosis leading to an exaggerated neointimal hyperplastic response or increased risk of platelet adhesion and scaffold thrombosis. Another concern of this device was that it required an 8-French guiding catheter. The PERSEUS study lead to the biodegradable peripheral Igaki-Tamai scaffolds to be used in Europe for peripheral cases [39].

### The REVA stent, a poly carbonate scaffold

The REVA scaffold (REVA Medical, Inc., San Diego, CA, USA) is a poly (iodinated desamino tyrosyl-tyrosine ethyl ester) carbonate device composed of iodinated-desaminotyrosinetyrosine. Following absorption, water, carbon dioxide, ethanol and iodinated-desaminotyrosinetyrosine are the end products from the Krebs cycle and excreted from the body. The REVA scaffold has no anti-proliferative drug coating and the bioresorption time is nearly 36 months. The slide and locking design prevented deformation and weakening of the polymer during scaffold deployment (Fig. 1). The radial force of the REVA scaffold has been reported to be greater than the MULTILINK BMS [40]. In the RESORB study, in which 27 patients with de novo lesions were enrolled, acute gain in lumen diameter and vessel shrinkage were satisfactory following device implantation. The mean diameter stenosis pre- and post implantation were 70 % and 5.9 % respectively. The pre-implantation and post-implantation lumen diameters were 0.88 ± 0.39 mm and 2.76 ± 0.36 mm, respectively. Despite these results, at 6-months follow-up LLL was 1.81 mm and TLR was 66.7 %, predominantly secondary to vessel recoil since the neo-intimal hyperplasia response was shown to be similar compared to BMS [41]. Following these findings, the scaffold has been redesigned and the second-generation ReZolve stent has stiff radiopaque polymer, a spiral ‘slide and lock’ mechanism and is coated with the antiproliferative drug sirolimus. In the RESTORE study, with 50 patients at 12 months follow-up, acute recoil was 3.8 ± 6.7 %, and LLL was 0.29 ± 0.33 mm at 12 months. At 6 months there were 2 MACE events in 12 patients [42]. Further improvements in the design of the scaffold have concluded in REVA’s current product. ReZolve2 is being tested in the Safety and Performance Study of the ReZolve2 Sirolimus-Eluting Bioresorbable Coronary Scaffold study (RESTORE-II) (*n* = 125) [43]. The company has presented a new clinical trial program named FANTOM investigating Fantom bioresorbable sccaffold with thinner strut thickness [44].

#### Poly salicylic acid stent: IDEAL BRS

The IDEAL BRS (Xenogenics Corp.; Canton, Massachusetts, United States) has a backbone made of polylactide anhydride mixed with a polymer of salicylic acid and sebacic acid. The backbone is coated with salicylate that controls the release of the antiproliferative drug sirolimus (8.3 μ/mm). With salicylate and sirolimus, the scaffold has potentially both anti-inflammatory and antiproliferative properties [45]. The IDEAL BRS was initially tested in humans (11 patients) in 2009. In this first experience, there was negligible neointimal suppression and a significant reduction in lumen area that was associated with problems relating to the dose release kinetics of sirolimus – namely that it was eluted too rapidly, with a surface area dose of only a quarter compared to Cypher drug eluting stent [46]. The new generation IDEAL BioStent device has been designed with a lower profile to aid delivery, as well as optimising the dose release kinetics of sirolimus. Preclinical studies of the IDEAL BioStent device are underway.

#### Myolimus-eluting Poly-L-Lactic acid scaffold: DESolve

The DESolve Myolimus-Eluting Bioresorbable Coronary Scaffold System has a poly L-lactic acid (PLLA) backbone and is coated with myolimus (3 mg/mm) - a sirolimus analogue. In porcine studies, the radial strength was sufficiently provided over a 3 month period, and the resorption phase was completed at up to 2-years [47]. In the multicentre DESolve-I FIM trial, which recruited 16 patients implanted with polylactide-based bioresorbable scaffold coated with bioabsorbable polymer eluting myolimus, the incidence of acute recoil was 6.4 % and the LLL was 0.19 mm at 6 months. Post-procedural IVUS analyses demonstrated a mean scaffold area 5.35 mm^2^ and a mean lumen area of 5.35 mm^2^. Six-month IVUS analyses did not significantly differ from the baseline IVUS with a mean scaffold area 5.61 mm^2^ and mean lumen area 5.10 mm^2^. Six-month optical coherence tomography (OCT) examination at follow-up demonstrated that 98.7 % of the struts to be covered by neointima. One-year clinical follow up demonstrated 3 MACE, 1 target vessel MI and 1 TLR; no patient was reported to have had a scaffold thrombosis [47]. In the multi-center, prospective DESolve Nx trial, 120 patients were treated with the DESolve Nx device - a PLLA-based polymer scaffold that is coated with novolimus (5 mg/mm), which is an active metabolite of sirolimus [48, 49]. Recruitment of patients in the trial has been completed and clinical follow-up is still on-going. DESolve Nx trial was successful in demonstrating the safety and efficacy of the DESolve scaffold, with a low 6-month LLL by QCA (0.20 ± 0.32 mm), low 6-month IVUS % volume obstruction (5 %), low 6-month neointimal hyperplasia (NIH) thickness by OCT (0.10 mm), sustained neointimal suppression through 18 months follow-up, low 24-month MACE rate (7.4 %), no reported late acquired incomplete strut apposition (ISA) by IVUS / OCT at 6 months and high percentage of strut coverage by OCT at 6 months (98.8 %) [48, 49]. The preclinical study for the next generation scaffold named DEsolve 100 with reduced strut thickness (100 μm) is ongoing.

#### Everolimus-eluting Poly-L-lactic acid scaffold (Absorb BVS)

The Abbott Vascular everolimus eluting bioresorbable vascular scaffold (ABSORB BVS) (Abbott Vascular, Santa Clara, CA, USA) has a backbone of PLLA, coated with layer of a 1:1 mixture of an amorphous matrix of poly-D, L-lactide (PDLLA) and an antiproliferative drug everolimus (8.2 μg/mm). The PDLLA controls the release of everolimus, 80 % of which is eluted at the end of the first month following implantation. The first version of Absorb BVS (Absorb BVS 1.0) had a strut thickness of 150 μm, a crossing profile of 1.4 mm, and constituted of circumferential out-of-phase zigzag hoops, with the struts linked directly together by thin and straight connections. In the first human study, ABSORB (*n* = 30), multimodality intravascular imaging including IVUS, IVUS-virtual histology (IVUS-VH), palpography and OCT were performed at 6-month and 2-years follow up. At 6-month clinical follow-up, there was only one ischemic driven major adverse event (non Q-wave myocardial infarction); in the following 42-months there were no reported MACE events [22, 50]. At the 4-year clinical follow-up there was no ST [51]. At 5-years the overall MACE event rate was 3.4 %. At 6-months follow-up LLL was 0.44 mm. The reduction in lumen area was 16.6 %, and the late recoil was 11.7 % [52]. The loss of radial strength with bioresorption, that was considered a consequence of scaffold shrinkage (6.94 ± 1.70 mm^2^ to 6.29 ± 1.47 mm^2^ at the 6 months follow-up), prompted the redesign of the scaffold. The re-designed Absorb BVS 1.1 had a strut design with in-phase hoops and straight links to provide additional radial support, and an updated polymer to provide additional mechanical strength for the scaffold [53]. The second generation ABSORB BVS was evaluated in the ABSORB Cohort B study. The studied population was divided into 2 groups; the first group (B1) had QCA, IVUS, IVUS palpography, IVUS-VH, IVUS echogenicity, and OCT at 6 months and 2 years. The second group (B2) had the same follow-up imaging processes at 1 and at 3 years. At 2 year clinical follow up overall MACE was 9.0 % [54]. In Cohort B1, IVUS analyses demonstrated the minimal lumen area to decrease during the 6-months follow-up (baseline: 6.60 ± 1.22 mm^2^ , 6-month:, *P* < 0.005), and to remain stable between 6-months and 2-years follow-up (6-month: 6.37 ± 1.12 mm^2^, 24-month: 5.99 ± 1.61 mm^2^, *P* = 0.26). On OCT evaluation, the scaffold area progressively increased (baseline: 7.47 ± 1.18 mm^2^, 6-months: 7.70 ± 1.34 mm^2^, 2-years 8.34 ± 1.83 mm^2^).

In Cohort B2, the mean scaffold area did not significantly change between post-implantation and 12-months in OCT and IVUS examinations. The vessel vasomotion was tested with the application of acetylcholine or methylergonovine and the lumen measurements during these tests elicited restoration of the vasomotion at 12 months after scaffold implantation [55]. At two years, intracoronary administration of nitrate was performed and a significant (*p* = 0.035) but modest (0.034 ± 0.09 mm) vasodilatation was demonstrated. At three years, the vasodilatation was improved (0.054 ± 0.12 mm, *p* = 0.005) [56]. Subsequently, preliminary results from the international, multi-center ABSORB EXTEND single arm study demonstrated an incidence of MACE of 7.3 %, ischemia driven TLR of 4.0 %, and stent thrombosis of 0.8 %, in 250 patients with 24 months of clinical follow-up [57].

ABSORB II constitutes the first randomized controlled trial comparing the efficacy and safety of a 2nd generation bioresorbable scaffold (Absorb, Abbott Vascular, Santa Clara, CA, USA) with a contemporary DES (Xience, Abbott Vascular, Santa Clara, CA, USA). The ABSORB II trial had a 2:1 single-masked design, recruiting 501 patients with stable and unstable angina symptoms to treatment with an everolimus eluting bioresorbable scaffold or a contemporary everolimus eluting metallic DES. The procedural details of the study were shown in Table 2. The co-primary endpoints of nitrate-induced vasomotion and changes in minimum lumen diameter (in-stent late loss) are to be reported at 3 years. Secondary outcomes recently reported at 1 year demonstrated no difference in major adverse cardiovascular events (defined as death, myocardial infarction or target lesion revascularization) between patients treated with a bioresorbable or a contemporary metallic DES (5 % vs. 3 %, *P* = 0.35). In addition, cumulative rates of first new or worsening angina were reported to be lower with the bioresorbable scaffold group compared to contemporary metallic DES (22 % vs. 30 %, *p* = 0.04), whereas the performance during maximum exercise and angina status by Seattle Angina Questionnaire were reported to be similar [57].

**Table 2:** Procedural details of ABSORB II trial

	Bioresorbable scaffold group (*n* = 335)	Metallic stent group (*n* = 166)	Difference (95 % CI)	*p*
Number of lesions	364	182		
Balloon dilatation prior to device implantation	364 (100 %)	180 (99 %)	1.10 % (−0.21, 3.92)	0.11
Planned overlap with the same type of device	56 (15 %)	20 (11 %)	4.40 % (−1.93, 9.94)	0.16
Additional implantation with the same device	14 (4 %)	11 (6.0)	−2.20 % (−6.91, 1.44)	0.25
More than one study device implanted	70 (19 %)	27 (15 %)	4.40 % (−2.57, 10.62)	0.21
Nominal size of study device (mm)	3.01 (0.31)	3.05 (0.28)	−0.04 (−0.10, 0.01)	0.10
Balloon dilatation after device implantation	221 (61 %)	107 (59 %)	1.92 % (−6.66, 10.67)	0.67
Nominal diameter of balloon used (mm)	3.08 (0.34)	3.16 (0.36)	−0.08 (−0.14, 0.01)	0.02
Maximum balloon pressure used (atm)	14.23 (3.43)	15.03 (3.33)	−0.80 (−1.4, −0.2)	0.01
Diameter of balloon used (mm)	3.29 (0.35)	3.35 (0.37)	−0.06 (−0.14, 0.02 )	0.15
Angiographic acute recoil of device following implantation per device (mm)	0.19 (0.19)	0.19 (0.18)	−0.00 (−0.04, 0.03)	0.85
Device success				
Clinical device success	361 (99 %)	182 (100 %)	−0.82 % (−2.39, 1.31)	0.55
Clinical procedural success	322 (96 %)	164 (99 %)	−2.68 % (−5.46, 0.80)	0.16

In ABSORB II, pre-procedure mean lumen area in the BVS and metallic stent groups were reported to be similar 4.84 ± 1.39 mm^2^ and 5.02 ± 1.47 mm^2^, respectively (*p* = 0.16). The post-procedure mean lumen area were 6.06 ± 1.44 mm^2^ and 6.85 ± 1.60 mm^2^ respectively (*p* < 0.001). Post-procedure acute gain in minimum lumen diameter was significantly larger in metallic stent group than in BRS group (1.46 ± 0.38 mm vs 1.15 ± 0.38 mm, respectively; *p* < 0.001). Post-procedure in-stent/in-scaffold diameter stenosis was larger in BRS group than in metallic stent group (16 ± 7 % vs 10 ± 5 %, respectively; *p* < 0.001). In post-procedure IVUS analyses, post-procedure mean lumen area was significantly less in BVS group than in metallic stent group (6.06 ± 1.44 mm^2^ vs 6.85 ± 1.60 mm^2^, respectively; *p* <0.001). Post-procedure minimal lumen area (5.73 ± 1.51 vs 4.89 ± 1.38, *p* <0.001) and post-procedural acute gain in minimal lumen area (3.60 ± 1.34 vs 2.85 ± 1.25, *p* <0.001) were higher in metallic stent group than in BVS group (Table 3). The incidence of definite scaffold thrombosis was 0.6 % in BRS and 0 % in metallic stent group (*p* = 1.0). At the end of the first year the incidence of MI was 15 (4 %) in the BRS group and 2 (1 %) patients in the metallic stent group (*p* = 0.06), and were mostly non Q-wave MI. There were two scaffold thrombosis, one within 24 h of implantation and the second on the 2nd day [57]. In the POLAR ACS registry [58], Absorb BVS was implanted in selected patients with unstable angina, non ST-elevated myocardial infarction (NSTEMI) and ST-elevated myocardial infarction (STEMI). 100 patients were followed up for 1 year with two MACE reported, namely periprocedural MI. At the very least this small registry demonstrated the potential feasibility of the Absorb BVS in the treatment of ACS [58].

**Table 3:** Angiographic and IVUS/IVUS-VH outcomes of ABSORB II trial

	Bioresorbable scaffold group (*n*=335)	Metallic stent group (*n*=166)	Difference (95 % CI)	*p*
Angiographic analysis				
Lesion length obstruction (mm)	13.8 (6.5)	13.8 (6.6)	0.00 (−1.18, 1.18)	1.00
Total scaffolded/stented length (mm)	21.1 (8.8)	20.9 (7.4)	0.24 (−1.17, 1.65)	0.74
Reference vessel diameter				
Pre-procedure diameter (mm)	2.59 (0.38)	2.63 (0.40)	−0.03 (−0.10, 0.04)	0.36
Postprocedure diameter (mm)	2.64 (0.36)	2.80 (0.34)	−0.16 (−0.22, −0.09)	<0.001
Minimum lumen diameter				
Pre-procedure diameter (mm)	1.07 (0.32)	1.05 (0.32)	0.02 (−0.03, 0.08)	0.44
Post-procedure in-stent or in-scaff old diameter (mm)	2.22 (0.33)	2.50 (0.33)	−0.28 (−0.34, −0.22)	<0.001
In-stent/in-scaff old acute gain (mm)	1.15±0.38	1.46±0.38	−0.30 (−0.37, −0.24)	<0.001
Diameter stenosis				
Pre-procedure percent diameter stenosis (%)	59±11 %	60±12 %	−1.07 (−3.11, 0.97)	0.30
Post-procedure in-stent/in-scaffold diameter stenosis (%)	16±7 %	10±5 %	5.37 (4.38, 6.36)	<0.001
Pre-procedural fibrotic tissue (%)	31.47±11.39	30.62±11.42	0.85 (−1.33, 3.04)	0.44
Pre-procedural fibrofatty tissue (%)	47.43±16.91	48.55±16.86	−1.12 (−4.35, 2.11)	0.50
Pre-procedural necrotic core (%)	16.20±6.86	16.15±6.90	0.05 (−1.27, 1.37)	0.94
Pre-procedural dense calcium (%)	4.90±4.73	4.68±4.10	0.22 (−0.61, 1.05)	0.60
Vessel area				
Pre-procedure area (mm^2^)	11.51±3.40	12.34±3.42	−0.83 (−1.47, −0.19)	0.02
Post-procedure area (mm^2^)	13.17±3.55	14.28±3.59	−1.11 (−1.78, −0.44)	0.001
Plaque area				
Pre-procedure plaque area (mm^2^)	6.67±2.52	7.30±2.68	0.6 (−1.12, 0.13)	0.01
Post-procedure plaque area (mm^2^)	7.11±2.46	7.43±2.44	−0.32 (−0.78, 0.14)	0.18
Mean lumen area				
Pre-procedure mean lumen area (mm^2^)	4.84±1.39	5.02±1.47	−0.19 (−0.47, 0.08)	0.16
Post-procedure mean lumen area (mm^2^)	6.06±1.44	6.85±1.60	−0.80 (−1.09, −0.50)	<0.001
Minimal lumen area				
Pre-procedure minimal lumen area (mm^2^)	2.04±0.72	2.13±0.83	−0.10 (−0.25, 0.05)	0.20
Post-procedure minimal lumen area (mm^2^)	4.89±1.38	5.73±1.51	−0.84 (−1.12, −0.57)	<0.001
Acute gain in minimal lumen area (mm^2^)	2.85±1.25	3.60±1.34	−0.75 (−0.99, −0.50)	<0.001

### Other bioresorbable scaffolds under clinical investigation

#### ART bioresorbable scaffold

The ART BRS (Arterial Remodeling Technologies; Noisy le Roi, France) is made from a PDLLA amorphous polymer. Notably the device does not contain an anti-proliferative drug. The device retains its structural integrity and scaffolding properties for a period of 5–7 months; the bioresorption ends within 18 months. In animal studies, there was no MACE reported and acute recoil rates were similar with BMS, with the mean lumen area and external elastic lamina area being increased at 9-months on IVUS evaluation [59, 60]. Based on these promising results, the Arterial Remodeling Transient Dismantling Vascular Angioplasty (ARTDIVA) [61] first in man trial (ClinicalTrials.gov Identifier: NCT01761578) was launched aiming to evaluate the safety and efficacy of the ART18Z bioresorbable scaffold in the treatment of patients with CAD [61]. In this trial 30 patients with a single de novo lesion were recruited in 5 medical centers in France. The mean diameter of reference vessel pre-procedure was 2.55 ± 0.30 mm, minimal luminal diameter was 0.99 ± 0.23 mm, the diameter stenosis was 61 ± 8 % and the lesion length was 7.54 ± 1.24 mm. At 6-months follow-up, in-stent diameter stenosis was 12 ± 7 % in-segment diameter stenosis was 17 ± 5 % and angiographic recoil was 4.3 %. During this follow-up period there was 1 ischemia driven TLR and 2 non-ischemia driven TLR, no MI and stroke/TIA [62].

#### Xinsorb BRS

The Xinsorb BRS (Huaan Biotechnology; Laiwu, China) is a fully bioresorbable sirolimus-eluting scaffold (strut thickness 160 μm) that consists of PLLA, polylactide- co-glycolide, and poly-L-lactide-co-e-caprolactone. 78 % of sirolimus is released from the Xinsorb BRS within 14 days [63].

In a comparison study between Xinsorb BRS and the Excel DES (JW Medical; Shandong, China) implanted in the coronaries of porcine models, there was no significant difference in percentage diameter stenosis (%DS) in the Xinsorb BRS compared to the Excel DES (18.6 % vs. 21.4 % at 30 days; *p* > 0.05 and 24.5 % vs. 27.7 % at 90 days; *p* > 0.05, respectively) [64]. At 3-month follow-up OCT imaging demonstrated significant red significant neointimal hyperplasia in porcine models. Subsequently the LLL and %DS were noticeably reduced. At 1-month follow-up, proximal, in-scaffold, and distal LLL of scaffold were 0.53 ± 0.41 mm, 0.68 ± 0.42 mm and 0.65 ± 0.24 mm, while the %DS were 9.5 ± 7.7 %, 17.6 ± 16.8 % and 10.5 ± 7.4 % respectively. At 3-months, proximal, in-scaffold, and distal LLL were 0.23 ± 0.48 mm, 0.77 ± 0.48 mm and 0.11 ± 0.35 mm, while %DS were 14.5 ± 9.4 %, 31.9 ± 13.6 % and 5.4 ± 3.6 % respectively. At 12-months, proximal, in-scaffold, and distal LLL were −0.13 ± 0.45 mm, 0.28 ± 0.41 mm and 0.18 ± 0.48 mm, while %DS were 2.4 ± 2.9 %, 14.1 ± 9.1 % and 8.6 ± 8.7 % respectively. At 18-month, proximal, in-scaffold, and distal LLL were 0.37 ± 0.57 mm, 0.09 ± 0.31 mm and −0.01 ± 0.41 mm, while %DS were 3.9 ± 4.6 %、13.7 ± 7.3 % and 6.9 ± 5.2 % respectively. Lumen area at 18-month was significantly larger than that at 3-month with a constant scaffold area [65]. In Xinsorb FIM trial (*n* = 30 patients), at 6-months follow-up, LLL was 0.18 ± 0.21 mm. In scaffolded segments, the diameter stenosis was 10.0 ± 4.2 % at post-implantation and 10.6 ± 6.6 % at 6-months follow-up (*p* = 0.70). At 6-months OCT follow-up (*n* = 19) the luminal area was 6.03 ± 0.76 mm^2^, scaffold area was 7.74 ± 0.62 mm^2^, in-scaffold area obstruction was 22.1 ± 6.1 %, neointimal thickness was 0.07 ± 0.04 mm with no thrombus detected. The 6-month IVUS follow-up revealed a mean vessel area 14.37 ± 0.90 mm^2^, mean neointimal area 3.11 ± 0.19 mm^2^, mean scaffold area 9.36 ± 0.21 mm^2^ and mean luminal area 6.26 ± 0.26 mm^2^ [66].

#### Mirage bioresorbable micro-fiber scaffold

Mirage Bioresorbable Micro-fiber Scaffold (Mirage BRMS, Manli Cardiology Singapore) is a PLLA-based sirolimus eluting scaffold. The device incorporates a helix coil design that provides high flexibility with a strut thickness of 125 μm in scaffolds with diameter ≤ 3 mm, and of 150 μm in scaffolds with diameter ≥ 3.5 mm. Mirage BRMS has a low crossing profile (0.044” – 0.058”), and relatively short bioresorption time (~14 months). Results of a porcine study were encouraging; namely no in-scaffold restenosis at 6-month follow-up, 99 % of the struts were covered while the mean NIH thickness on top of covered struts was 0.08 ± 0.03 mm at 6-month follow-up [67]. Frequency of covered and uncovered struts per lesion were 99.85 ± 0.33 % and 0.15 ± 0.33 % respectively. The frequency of malapposed struts per lesion was 0.03 ± 0.08 %, and malapposition strut-to-lumen distance was 0.28 mm (there was only one malapposed strut at 6-month follow-up). In QCA analysis, MLD and % DS was 2.34 ± 0.49 mm and 2.13 ± 0.47 mm, 17.1 ± 11.4 % and 22.8 ± 15.0 %, at post-procedure and at 6-months, respectively. At 6-months, LLL was 0.21 ± 0.20 mm and late recoil was 0.16 ± 0.12 mm. Both in-scaffold and in-segment angiographic binary restenosis ratios were 0 % at 6-month [67]. Patient enrolment in FIM trial was completed in September 2014 and the results are expected to be presented at the end of 2015.

## Conclusion

For the last 20 years percutaneous coronary revascularization has evolved, with the current premise that stent implantation to be the standard of care in appropriately selected patients [68]. Considering that coronary stenting with metallic devices may results in persistent inflammation and endothelial dysfunction, an issue that has been reduced but not eliminated with newer generation DES [69], the temporary scaffold that would safeguard vessel patency and then it would disappear appears as the ideal solution for treating CAD [70]. These devices at the very least have to provide comparable performances to contemporary DES in the short term, with the potential promise of enhanced longer term benefits due to freeing the vessel wall from the metallic cage and allowing the vessel to potentially restore its vascular function (vessel vasomotion) adaptive shear stress and would permit late luminal enlargement, and late expansive remodelling. Ongoing, and future randomized trials assessing the efficacy of the multitude of bioresorbable scaffolds – currently 16 different scaffolds are being developed and under investigation – will ultimately determine the clinical value of this fourth revolution in interventional cardiology.
